# Supplementary material for: Deep learning approach to describe and classify fungi microscopic images
Source: PLoS One. 2020 Jun 30;15(6):e0234806. doi: 10.1371/journal.pone.0234806 (PMC7326179; doi:10.1371/journal.pone.0234806)
Supplement: S1 Appendix — (PDF) [file pone.0234806.s001.pdf]

S1 Appendix

To make this paper self-contained, below, we recall definitions of Bag of Words [24] and Fisher Vector [25].

Bag of Words. Let $\{X_i\}_{i=1}^N$ be the representations of N training images obtained from the last convolutional layer of CNN, where $X_i = \{x_{i,j} \in \mathbb{R}^{c_n}\}_{j=1}^{w_n h_n}$, while w_n, h_n, and c_n are dimensions of the nth CNN layer. The representations are consolidated $X = X_1 \cup X_2 \cup \ldots \cup X_N$, and a codebook of size K is calculated using k-means clustering on X. Let $\{\mu_k \in \mathbb{R}^{c_n}, k = 1, \ldots, K\}$ denote the centers of the obtained clusters. Moreover, let us denote $NN(x_{i,j})$ as the index of the cluster center nearest to $x_{i,j}$:

$$NN(x_{i,j}) = k \ : \ d(x_{i,j}, \mu_k) \leq d(x_{i,j}, \mu_l) \text{ for all } l \in \{1, \ldots, w_n h_n\}.$$

The Bag of Words counts the number of points from X_i, which are closer to particular clusters:

$$BoW(X_i) = (card\{x_{i,j} \in X_i \ : \ NN(x_{i,j}) = k\})_{k=1,\ldots,K}.$$

Fisher Vector. Similarly to Bag of Words, the Fisher Vector starts with consolidating the representations into X. Then, X is used to generate the Gaussian Mixture Model (GMM) $\lambda = \{\pi_k, \mu_k, \Sigma_k, k = 1, \ldots, K\}$, where π_k, μ_k and Σ_k denote the weight, mean vector and covariance matrix of kth Gaussian, and K is the number of Gaussians. Intuitively, the Fisher Vector characterizes a particular representation X_i with a gradient vector derived from GMM. More formally, let $\mathcal{L}(X_i|\lambda) = log\, p(X_i|\lambda)$ is the likelihood that point $x_{i,j}$ was generated by the GMM (under the independence assumption):

$$\mathcal{L}(X_i|\lambda) = log\ \Pi_{x_{i,j} \in X_i} p(x_{i,j}|\lambda)) = \sum_{x_{i,j} \in X_i} log\ p(x_{i,j}|\lambda),$$

where:

$$p(x_{i,j}|\lambda) = \sum_{k=1}^{K} \pi_k p_k(x_{i,j}|\lambda).$$

Assuming that the covariance matrices are diagonal (for ease of calculation), the derivations $\frac{\partial \mathcal{L}(X_i|\lambda)}{\partial \mu_k^d}$ and $\frac{\partial \mathcal{L}(X_i|\lambda)}{\partial \sigma_k^d}$ (where $\sigma_k^d = diag(\Sigma_k)$ and superscript d denotes the dth dimension of a vector) can be effectively computed as [25]:

$$\frac{\partial \mathcal{L}(X_i|\lambda)}{\partial \mu_k^d} = \sum_{x_{i,j} \in X_i} \gamma_k(x_{i,j}) \left[\frac{x_{i,j}^d - \mu_k^d}{(\sigma_k^d)^2} \right],$$

$$\frac{\partial \mathcal{L}(X_i|\lambda)}{\partial \sigma_k^d} = \sum_{x_{i,j} \in X_i} \gamma_k(x_{i,j}) \left[\frac{(x_{i,j}^d - \mu_k^d)^2}{(\sigma_k^d)^3} - \frac{1}{\sigma_k^d} \right],$$

where $\gamma_k(x_{i,j})$ is the soft assignment of $x_{i,j}$ to kth Gaussian:

$$\gamma_k(x_{i,j}) = p(k|x_{i,j}, \lambda) = \frac{\pi_k p_k(x_{i,j}|\lambda)}{\sum_{l=1}^{K} \pi_l p_l(x_{i,j}|\lambda)},$$

The gradient vector is a concatenation of the partial derivatives with respect to all the parameters. To normalize the dynamic range of dimensions, diagonal of the Fisher information matrix F_λ is computed as:

$$F_\lambda = E_{X_i}[\nabla_\lambda \mathcal{L}(X_i|\lambda) \nabla_\lambda \mathcal{L}(X_i|\lambda)'],$$

and then applied to partial derivatives, resulting in the final definition of the Fisher vector:

$$FV(X_i) = \left(f_{\mu_k^d}^{-1/2} \frac{\partial \mathcal{L}(X_i|\lambda)}{\partial \mu_k^2}, f_{\sigma_k^d}^{-1/2} \frac{\partial \mathcal{L}(X_i|\lambda)}{\partial \sigma_k^d} \right)_{k=1..K},$$

where $f_{\mu_k^d}$ and $f_{\sigma_k^d}$ are the corresponding terms on the diagonal of F_λ.
