# Supplementary material for: Deep learning approach to describe and classify fungi microscopic images
Source: PLoS One. 2020 Jun 30;15(6):e0234806. doi: 10.1371/journal.pone.0234806 (PMC7326179; doi:10.1371/journal.pone.0234806)
Supplement: S2 Appendix — (PDF) [file pone.0234806.s002.pdf]

S2 Appendix

In this section, we describe our Digital Images of Fungus Species database (DIFaS). S1 and S2 Fig present images of the first and the second preparation together with their foreground-background masks. Moreover, we present the number of foreground and background patches generated using the sliding window with 128-pixels shift (the number of patches overlapped by less than 50%). They are presented in S1 and S2 Tables for images from S1 Fig, and in S3 and S4 Tables for images from S2 Fig.
