# Supplementary material for: Deep learning approach to describe and classify fungi microscopic images
Source: PLoS One. 2020 Jun 30;15(6):e0234806. doi: 10.1371/journal.pone.0234806 (PMC7326179; doi:10.1371/journal.pone.0234806)
Supplement: S1 Table — (PDF) [file pone.0234806.s003.pdf]

S1 Table. The number of foreground patches overlapped by less than 50% for the images from S1 Fig.

Strain	Image 1	Image 2	Image 3	Image 4	Image 5	Image 6	Image 7	Image 8	Image 9	Image 10	Total
CA	3	5	3	4	3	5	2	9	11	12	57
CG	185	60	151	131	170	43	129	118	26	57	1070
CL	8	18	26	10	4	19	9	3	14	30	111
CN	4	3	2	3	11	3	4	1	4		35
CP	67	92	36	39	109	29	53	38	161	83	707
CT	39	3	5	113	58	41	8	30	17	17	331
MF	4	6	3	5	1	4	2	2	4	6	37
SB	55	191	102	45	60	61	33	79	9	204	839
SC	27	5	7	11	22	4	5	8	9	9	107
