# Supplementary material for: Deep learning approach to describe and classify fungi microscopic images
Source: PLoS One. 2020 Jun 30;15(6):e0234806. doi: 10.1371/journal.pone.0234806 (PMC7326179; doi:10.1371/journal.pone.0234806)
Supplement: S2 Table — (PDF) [file pone.0234806.s004.pdf]

S2 Table. The number of background patches overlapped by less than 50% for the images from S1 Fig.

Strain	Image 1	Image 2	Image 3	Image 4	Image 5	Image 6	Image 7	Image 8	Image 9	Image 10	Total
CA	241	216	237	241	248	236	236	187	204	186	2232
CG	14	79	0	52	14	9	28	0	43	70	309
CL	174	66	109	88	144	92	142	156	88	57	1116
CN	240	252	243	238	228	236	256	238	245		2176
CP	48	43	68	32	5	104	9	41	3	9	362
CT	83	183	98	9	44	46	103	52	117	79	814
MF	230	135	198	196	226	211	212	181	194	147	1930
SB	88	6	51	27	79	33	142	19	222	0	667
SC	45	213	179	127	126	154	104	184	219	170	1521
