# Supplementary material for: Deep learning approach to describe and classify fungi microscopic images
Source: PLoS One. 2020 Jun 30;15(6):e0234806. doi: 10.1371/journal.pone.0234806 (PMC7326179; doi:10.1371/journal.pone.0234806)
Supplement: S3 Table — (PDF) [file pone.0234806.s005.pdf]

S3 Table. The number of foreground patches overlapped by less than 50% for the images from S2 Fig.

Strain	Image 1	Image 2	Image 3	Image 4	Image 5	Image 6	Image 7	Image 8	Image 9	Image 10	Total
CA	5	2	3	3	8	5	8	10	7	4	55
CG	103	171	132	176	131	92	18	51	8	8	890
CL	28	28	6	7	8	5	3	21	9	7	122
CN	3	3	2	7	7	4					26
CP	107	72	109	144	63	57	59	12	5	7	635
CT	6	27	21	13	21	15	3	10	9	3	128
MF	3	2	2	6	3	2	4	2	3	2	29
SB	60	2	6	9	21	71	8	12	10	49	248
SC	7	16	14	3	11	9	7	10	3	14	94
