# Supplementary material for: Deep learning approach to describe and classify fungi microscopic images
Source: PLoS One. 2020 Jun 30;15(6):e0234806. doi: 10.1371/journal.pone.0234806 (PMC7326179; doi:10.1371/journal.pone.0234806)
Supplement: S4 Table — (PDF) [file pone.0234806.s006.pdf]

S4 Table. The number of background patches overlapped by less than 50% for the images from S2 Fig.

Strain	Image 1	Image 2	Image 3	Image 4	Image 5	Image 6	Image 7	Image 8	Image 9	Image 10	Total
CA	215	229	228	224	235	204	203	204	227	202	2171
CG	1	5	31	0	17	2	59	70	162	187	534
CL	54	54	179	77	194	158	230	74	57	112	1189
CN	241	252	253	220	243	243					1452
CP	2	2	30	13	27	10	50	63	138	189	524
CT	108	96	110	99	89	84	171	90	168	192	1207
MF	158	227	225	166	204	185	179	186	171	189	1890
SB	69	176	227	188	185	47	220	190	215	111	1628
SC	144	122	111	188	131	176	248	190	174	195	1679
