# Supplementary figures and images for: Deep learning approach to describe and classify fungi microscopic images
Source: PLoS One. 2020 Jun 30;15(6):e0234806. doi: 10.1371/journal.pone.0234806 (PMC7326179; doi:10.1371/journal.pone.0234806)

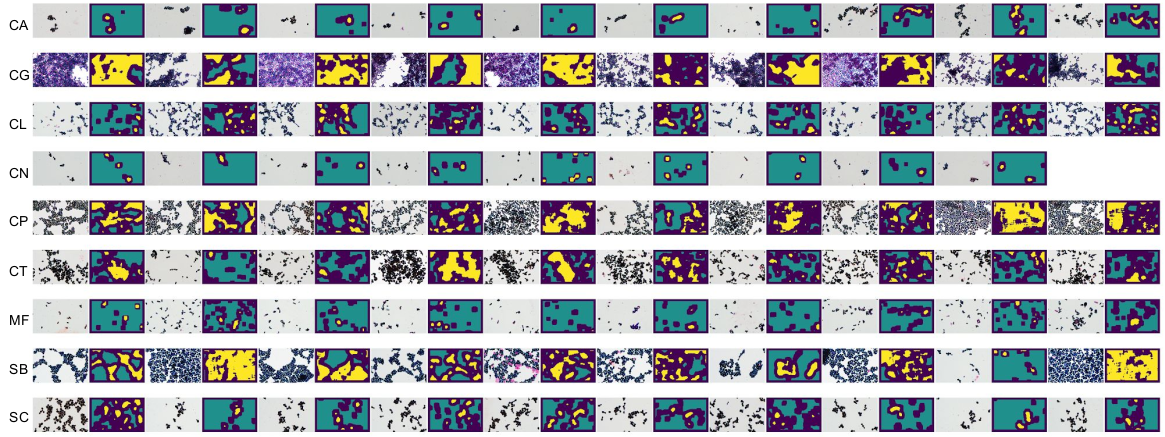

Supplement: S1 Fig — (TIF) [file pone.0234806.s007.tif]

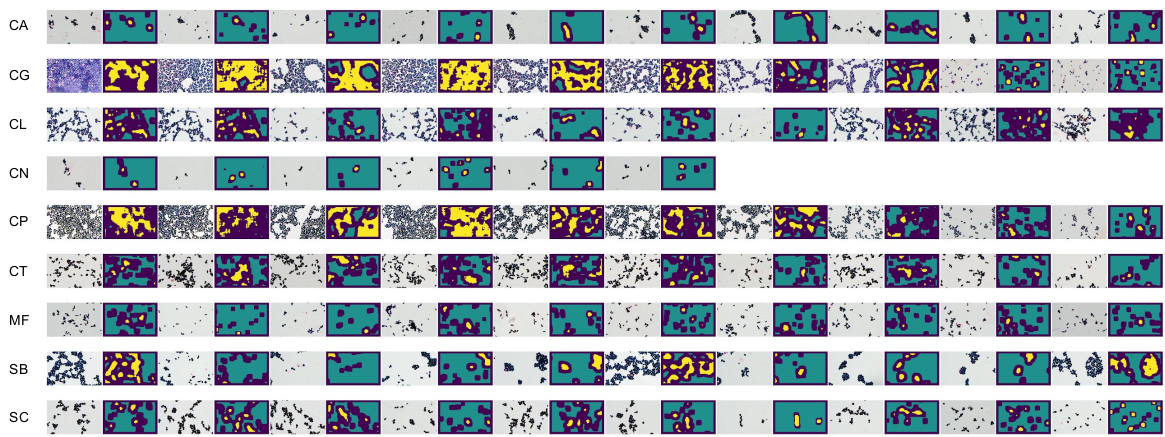

Supplement: S2 Fig — (TIF) [file pone.0234806.s008.tif]
